# Genome-wide DNA polymorphisms in two cultivars of mei (*Prunus mume* sieb. et zucc.)

**DOI:** 10.1186/1471-2156-14-98

**Published:** 2013-10-06

**Authors:** Lidan Sun, Qixiang Zhang, Zongda Xu, Weiru Yang, Yu Guo, Jiuxing Lu, Huitang Pan, Tangren Cheng, Ming Cai

**Affiliations:** 1 Beijing Key Laboratory of Ornamental Plants Germplasm Innovation and Molecular Breeding, National Engineering Research Center for Floriculture, College of Landscape Architecture, Beijing Forestry University, 100083 Beijing, P.R. China; 2 BGI-Shenzhen, 518083 Shenzhen, P.R. China

**Keywords:** Low-depth genome sequencing, SNPs, InDels, SSRs, SNP array

## Abstract

**Background:**

Mei (*Prunus mume* Sieb. et Zucc.) is a famous ornamental plant and fruit crop grown in East Asian countries. Limited genetic resources, especially molecular markers, have hindered the progress of mei breeding projects. Here, we performed low-depth whole-genome sequencing of *Prunus mume* ‘Fenban’ and *Prunus mume* ‘Kouzi Yudie’ to identify high-quality polymorphic markers between the two cultivars on a large scale.

**Results:**

A total of 1464.1 Mb and 1422.1 Mb of ‘Fenban’ and ‘Kouzi Yudie’ sequencing data were uniquely mapped to the mei reference genome with about 6-fold coverage, respectively. We detected a large number of putative polymorphic markers from the 196.9 Mb of sequencing data shared by the two cultivars, which together contained 200,627 SNPs, 4,900 InDels, and 7,063 SSRs. Among these markers, 38,773 SNPs, 174 InDels, and 418 SSRs were distributed in the 22.4 Mb CDS region, and 63.0% of these marker-containing CDS sequences were assigned to GO terms. Subsequently, 670 selected SNPs were validated using an Agilent’s SureSelect solution phase hybridization assay. A subset of 599 SNPs was used to assess the genetic similarity of a panel of mei germplasm samples and a plum (*P. salicina*) cultivar, producing a set of informative diversity data. We also analyzed the frequency and distribution of detected InDels and SSRs in mei genome and validated their usefulness as DNA markers. These markers were successfully amplified in the cultivars and in their segregating progeny.

**Conclusions:**

A large set of high-quality polymorphic SNPs, InDels, and SSRs were identified in parallel between ‘Fenban’ and ‘Kouzi Yudie’ using low-depth whole-genome sequencing. The study presents extensive data on these polymorphic markers, which can be useful for constructing high-resolution genetic maps, performing genome-wide association studies, and designing genomic selection strategies in mei.

## Background

Mei (*Prunus mume* Sieb. et Zucc., 2n=2x=16) is a member of Rosaceae, sub-family Prunoideae [1]. It originated in southwestern China, and has been cultivated in China for more than 3000 years [1]. Presently, it is also widely cultivated in other East Asian countries such as Japan and Korea [1,2]. Mei blossoms possess many conspicuous ornamental characteristics, such as vibrantly colored corollas and various types of flowers. Mei is characterized by an inherent tolerance to low temperatures (−4 to −2°C), which allows this species to flower in winter or early spring when most other ornamental plants are still dormant [1,2]. Therefore, it has been widely cultivated as an early-blooming garden ornamental plant. Mei can also be converted into many useful products, including salted mei, mei wine, and juice, which are considered to have important nutritional and medicinal value [2]. All of the above mentioned three products are extensively consumed in East Asian countries [2]. There is an urgent need to cultivate new mei varieties with enhanced ornamental and nutritional value, suitable for consumer needs. However, traditional mei breeding is relatively cumbersome, tedious, and time-consuming. This is mainly because mei is a woody perennial that takes a long time to reach its reproductive age. Recently, DNA markers have been used to analyze genetic diversity, distinguish varieties, and construct genetic maps [3-6]. However, quantitative trait locus (QTL) analysis, genome-wide association studies (GWAS), and genomic selection studies are impeded due to the limited availability of sufficient DNA markers.

With the advent of NGS technologies, entire genomes have been sequenced more efficiently and economically than ever before. The alignment of the short reads obtained from different varieties of mei, to the reference genome, has provided the perfect opportunity to identify a large number of polymorphic DNA markers in parallel, including SNPs, InDels, and SSRs, which are well known in crop species such as rice [7], eggplant [8], watermelon [9], and Chinese cabbage [10]. However, the heterozygous complexity of the genome of ornamental plants and the cost of whole genome deep-coverage sequencing are limiting factors in the genome-wide identification of DNA polymorphisms using massively parallel sequencing technology. Recently, the availability of the mei shotgun genome assembly [5], which was completed using the Solexa platform, facilitated the discovery of massive numbers of polymorphic DNA markers and the identification of genome-wide variants.

SNPs, InDels, and SSRs are important DNA markers due to their abundance, stability, codominance, efficiency, and ready automation. They have been widely useful for analysing genetic diversity, constructing high-density genetic maps, performing GWAS, and designing genomic selection strategies in many organisms [9,11-14]. For example, high-resolution genetic map have been constructed to anchor the assembly sequences of watermelon using SSRs, InDels, and SVs, all found using whole-genome resequencing [9]. An initial map of human InDel variation was constructed using DNA resequencing traces to identify polymorphisms that can influence human diseases [12]. One study on GWAS in maize indicated that SNPs can be associated with a phenotype ascribed to linkage disequilibrium (LD) [13]. Recently, a genetic map containing 1,484 SNP markers was constructed using RAD strategy in a segregating F_1_ population derived from *Prunus mume* ‘Fenban’ and *Prunus mume* ‘Kouzi Yudie’ which anchored 83.9% assembly sequences of mei genome [5]. However, the remaining 16.1% assembly sequences of mei genome have not been anchored. These SNPs were distributed unevenly across each chromosome, suggesting that some regions had fewer SNPs than others [5].

In the present study, we obtained a large number of putative polymorphic markers including SNPs, InDels and SSRs between ‘Fenban’ and ‘Kouzi Yudie’ by using low-depth genome sequencing of the two mei cultivars. We also identified the frequency and distribution of these markers in different regions of eight mei pseudo-chromosomes. In addition to the validation of the SNPs using Agilent SureSelect liquid-based hybrid capture system, InDels and SSRs were also partially validated by actual use as DNA markers. The information described here can be used to construct fully integrated maps of natural genetic variation that include SNPs, InDels, and SSRs. The maps can be used to identify polymorphisms that directly influence mei phenotypes. This information permits novel observations that can be used in mei genetics and breeding projects.

## Results and discussion

### Sequence mapping and detection of polymorphic DNA markers

**Figure 1:**
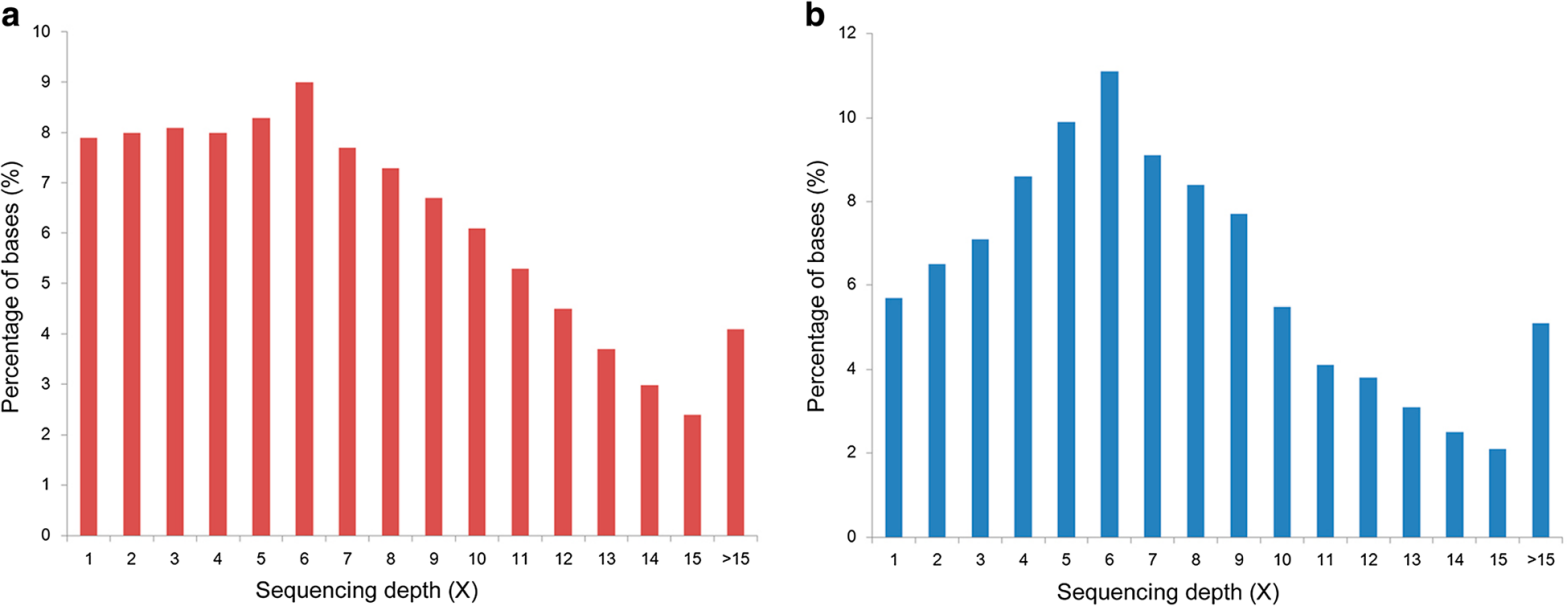
Sequence depth distribution of ‘Fenban’ (a) and ‘Kouzi Yudie’ (b).

Low-depth whole-genome sequencing of *Prunus mume* ‘Fenban’ and *Prunus mume* ‘Kouzi Yudie’ was performed using Illumina Genome Analyzer (GA) II instruments [5]. About 2.2 Gb of sequencing filtered data for ‘Fenban’ and ~2.3 Gb of data for ‘Kouzi Yudie’ were then aligned to the mei reference genome using BWA software [15]. About 2.0 Gb and ~2.1 Gb of sequencing filtered data were successfully mapped to the mei reference genome. A total of 1464.1 Mb and 1422.1 Mb of sequencing data were uniquely mapped to the mei reference genome and translated into ~6-fold coverage of the mei assembly sequences (237 Mb), respectively (Figure 1) [5]. Ultimately, we identified a large set of putative polymorphic DNA markers in the shared 196.9 Mb of the two cultivar sequence datasets. They covered 83.1% of the mei assembly sequences (~237 Mb).

The putative polymorphic markers were classified into three types: SNPs, in which a single nucleotide was altered at a specific location in one of the two cultivars [16]; InDels, in which one phenotype had a stretch of nucleotides not present in the other [16]; and SSRs, in which repeat motifs showed different lengths in the two cultivars. Using fairly stringent criteria (see Methods), we identified 200,627 SNPs, 4900 InDels, and 7,063 SSRs in the two cultivars (Additional files 1, 2, 3), and 89.2% SNPs, 90.8% InDels, and 86.9% SSRs were identified in eight pseudo-chromosomes of the mei genome (Table 1). The average densities of these markers were 899 SNPs/Mb, 22 InDels/Mb, and 31 SSRs/Mb in the eight pseudo-chromosomes. These markers, which were found in the pseudo-chromosomes, were used to increase the resolution of the genetic map based on the ‘Fenban’ and ‘Kouzi Yudie’ F_1_ segregating population. This map was constructed using the previously described RAD strategy [5]. About 83.9% of the assembled sequences were anchored to eight pseudo-chromosomes of the mei genome using the genetic map [5]. Hence, the remaining markers (21,755 SNPs, 452 InDels, and 928 SSRs), which were not detected in the pseudo-chromosomes, will be used to anchor other assembled sequences in the near future.

**Table 1:** Distribution of polymorphic DNA markers present in both ‘Fenban’ and ‘Kouzi Yudie’ on eight mei pseudo-chromosomes

**Pseudo-chromosome**	**No. of SNPs**	**No. of InDels**	**No. of SSRs**	**Physical size (Mb)**
Pseudo-chromosome 1	25,395 (941)	596 (22)	798 (30)	26.8
Pseudo-chromosome 2	40,350 (961)	895 (21)	1,376 (33)	42.1
Pseudo-chromosome 3	27,111 (1,084)	548 (22)	764 (31)	24.6
Pseudo-chromosome 4	20,975 (874)	552 (23)	789 (33)	24.0
Pseudo-chromosome 5	20,446 (786)	545 (21)	760 (29)	25.8
Pseudo-chromosome 6	19,016 (906)	516 (25)	584 (27)	21.3
Pseudo-chromosome 7	14,219 (836)	344 (20)	562 (33)	17.1
Pseudo-chromosome 8	11,360 (668)	452 (27)	502 (29)	17.3
Total	178,872 (899)	4,448 (22)	6,135 (31)	199.0

Note: The numbers in parentheses indicate the mean numbers of SNPs, InDels, and SSRs detected per 1 Mb genome sequence.

The number of polymorphic DNA markers varied across each pseudo-chromosome. The highest number of SNPs (40,350) and SSRs (1,376) was observed in pseudo-chromosome 2. This was 3.6-fold higher than the number of SNPs (11,360) found in pseudo-chromosome 8 and 2.7-fold higher than the number of SSRs (502) in pseudo-chromosome 8, which had the fewest SNPs and SSRs. The highest number of InDels (895) was observed in pseudo-chromosome 2. This was 2.6-fold more than the number of InDels (344) detected in pseudo-chromosome 7, which had the fewest (Table 1). The marker distribution of individual pseudo-chromosomes was uneven, as in rice [7]. This result can be attributed to the variations in chromosome size in the mei genome. Pseudo-chromosome 2 was found to be 42.1 Mb in size, which was 2.5-fold the size of pseudo-chromosome 7 (17.1 Mb) and was 2.4-fold that of pseudo-chromosome 8 (17.3 Mb) (Table 1).

**Figure 2:**
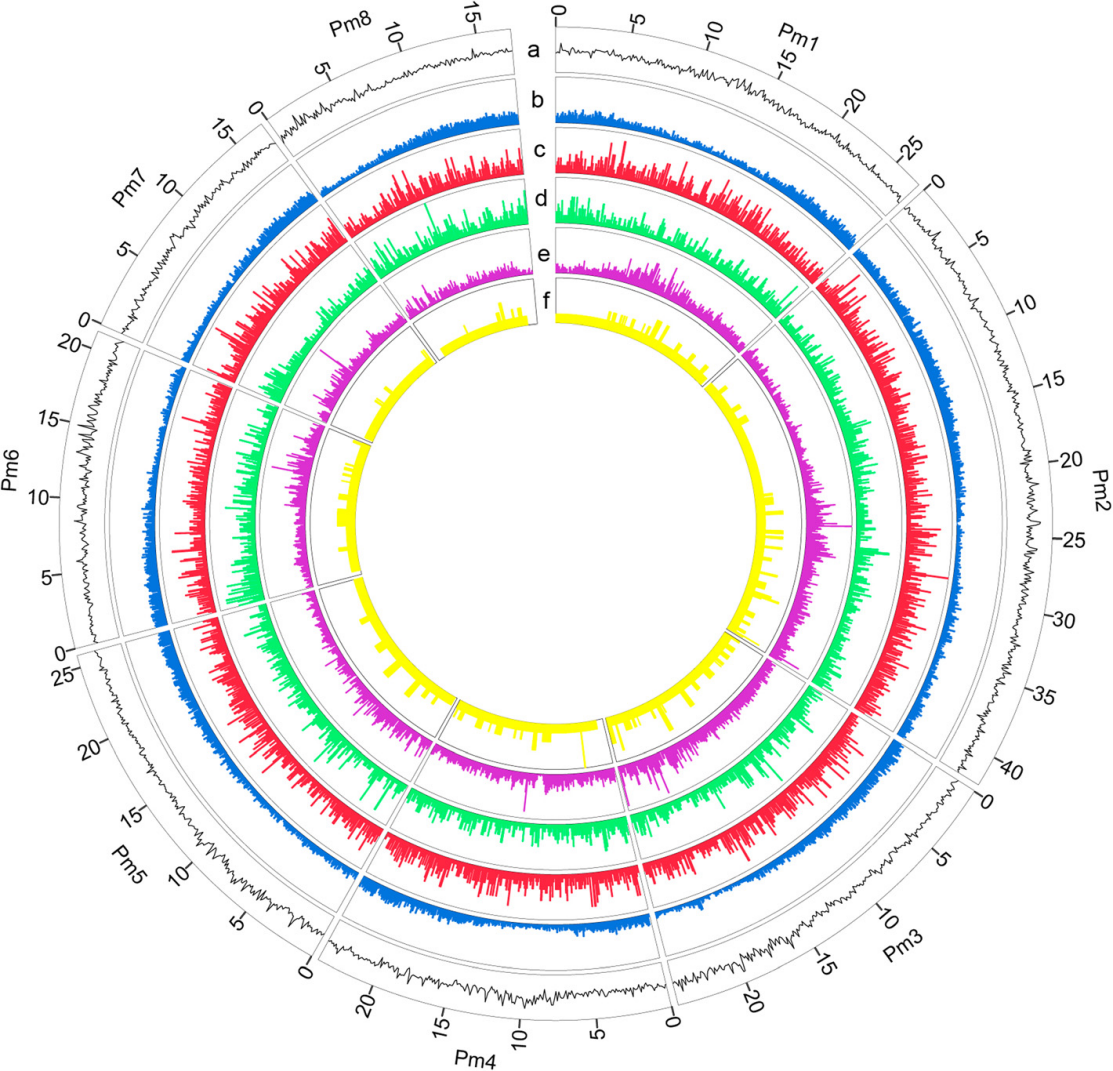
**Distribution of polymorphic DNA markers between ‘Fenban’ and ‘Kouzi Yudie’ in the mei pseudo-chromosomes.** All tracks are plotted in 100 Kb windows. The y axis ranges from 0 to 100%. **(a)** GC content shown in black; **(b)** Gene density shown in blue; **(c)** SSR density shown in red; **(d)** InDel density shown in cyan; **(e)** SNP density shown in purple; **(f)** Numbers of SNPs validated in a SNP array, shown in yellow.

The average density of these markers was also different in each pseudo-chromosome. We calculated the number of these markers within a 0.1 Mb sliding window across the genome to compare their distribution and frequency in each pseudo-chromosome (Figure 2). The distribution of polymorphic DNA markers was not homogeneous within pseudo-chromosomes. This was especially true of the distribution of SNPs. For example, 58 high-density regions with > 1000 SNPs/Mb, and 12 low-density regions with < 500 SNPs/Mb were identified in mei pseudo-chromosomes (Figure 2 and Additional file 1). All pseudo-chromosomes except pseudo-chromosome 8 were found to have regions with several markers, and regions in which these markers were scarce. For example, on pseudo-chromosome 2, the region from 27 Mb to 28 Mb contained 2,123 SNPs, 34 InDels, and 50 SSRs, but the region from 15 Mb to 16 Mb had only 488 SNPs, 15 InDels, and 17 SSRs (Additional files 1, 2, 3). We found that these markers were more common in intergenic regions than in coding sequence (CDS) regions (Figure 2 and Additional files 1, 2, 3). This result was consistent with those reported in previous studies in rice [7,17] and maize [18]. The uneven distribution of markers in different parts of the genome could be ascribed to the functional importance of these markers in CDS regions, which experience more negative selective pressure than intergenic regions [19].

### Annotation of SNPs, InDels, and SSRs

A total of 200,627 SNPs, 4,900 InDels, and 7,063 SSRs were annotated using the Mei Annotation Project Database release (http://prunusmumegenome.bjfu.edu.cn). The polymorphic markers showed only minimal distribution in CDS regions (Additional files 1, 2, 3). Only 38,773 SNPs (19.3% of the total), 174 InDels (3.6% of the total), and 418 SSRs (5.9% of the total) were distributed in the 22.4 Mb CDS region (Additional files 1, 2, 3). There were more SNPs than InDels or SSRs in CDS regions. This difference can be explained by the fact that InDels and SSRs are more deleterious than SNPs in CDS regions, as indicated by InDels and SSRs that cause frame shift mutations and amino acid substitutions that have major changes to gene function [19,20]. However, SNPs often produce synonymous mutations that have little or no impact on gene function [21]. In our study, among the 38,773 SNPs, 28,020 SNPs were synonymous and 10,753 SNPs were nonsynonymous. The ratio of nonsynonymous to synonymous substitutions was 0.38, which is lower than that of Arabidopsis (0.83) [22], rice (1.29) [17], and soybean (1.61) [23]. It is possible that this difference have been caused by strong purifying selection at nonsynonymous sites of SNPs in CDS regions of mei. However, a more convincing explanation is essential with increasing recognition of mei as a study material for woody plants.

**Figure 3:**
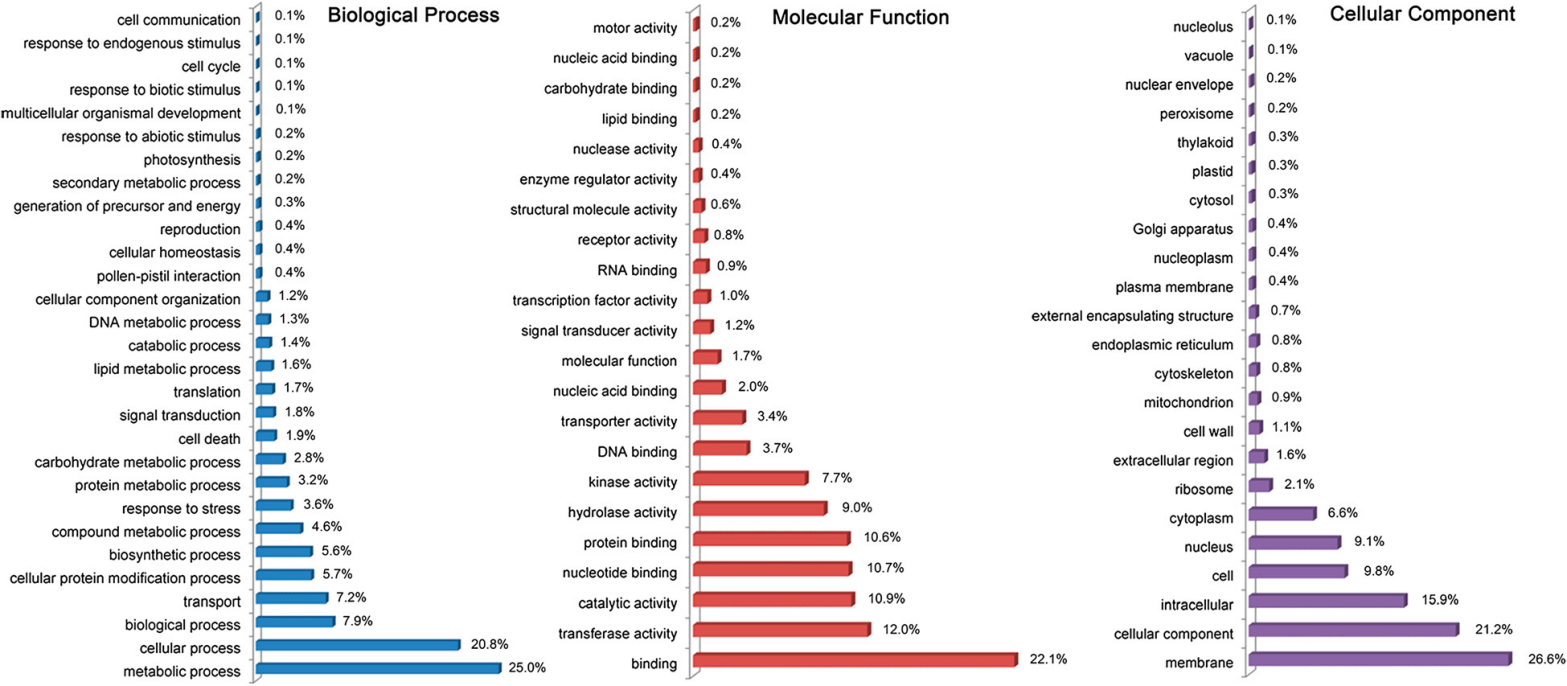
GO term representation (%) of 9,557 CDS containing DNA polymorphic markers.

Despite the relatively low abundance, 63.0% (9,557 in total) of these marker-containing CDS sequences were assigned to one or more functional annotations [Gene ontology (GO) terms] [8]. These annotations covered all the three top-level categories, specifically biological process, cellular component, and molecular function. There were 17,148 GO terms associated with biological process, 5,204 with cellular component, and 22,586 with molecular function (Figure 3 and Additional file 4). Among biological process ontology, metabolic process (25.0%) and cellular process (20.8%) formed the largest categories. Under the cellular component ontology, the major proportion of terms fell into the membrane (26.6%) category. However, 11,424 (50.6%) genes of the molecular function ontology were involved in binding activity (Figure 3). The present study provides a large set of polymorphic markers associated with functional genes and our results may facilitate MAS-directed breeding in mei.

### Use of SNP markers on arrays

**Figure 4:**
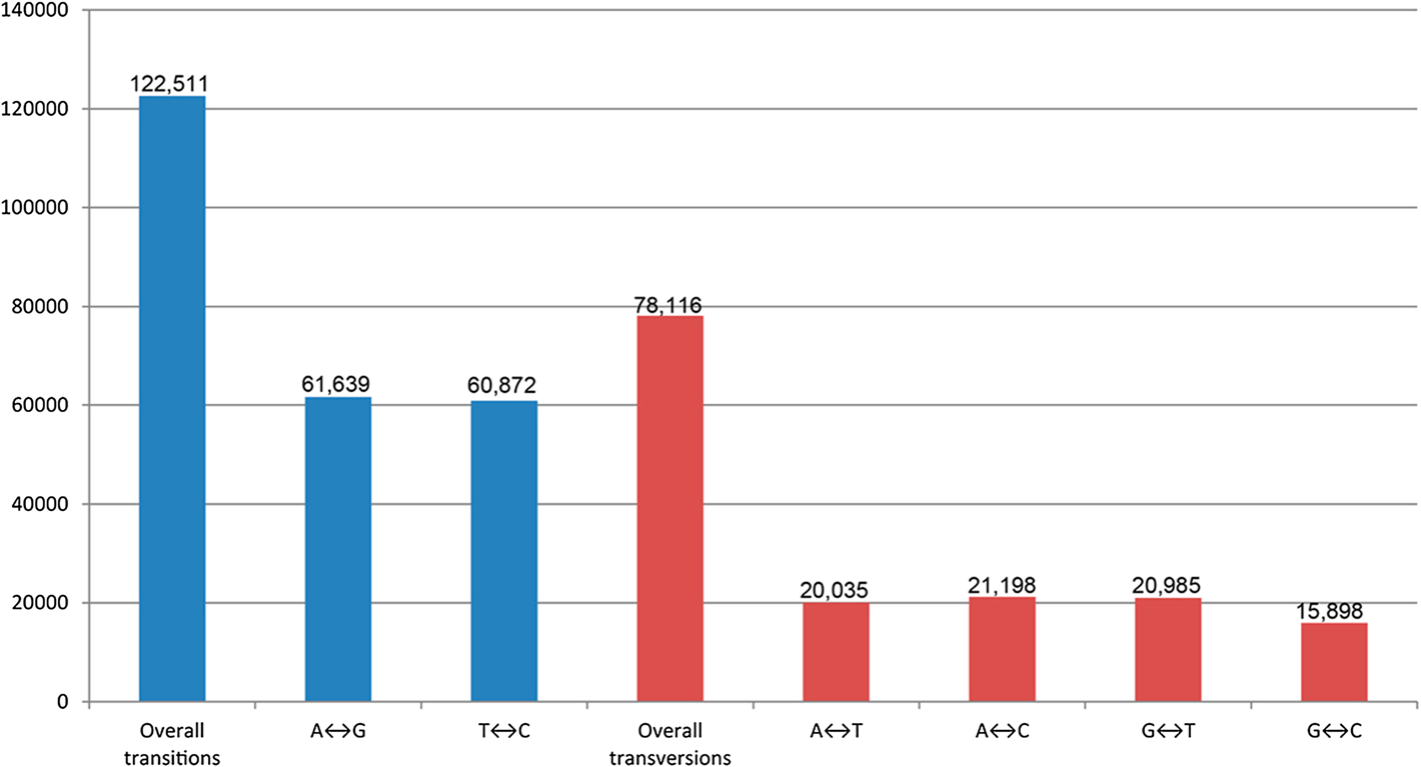
Transitions and transversions occurring within a set of 200,627 SNPs in mei.

Whole-genome sequencing allowed us to detect 200,627 candidate SNP markers in ‘Fenban’ and ‘Kouzi Yudie’. The density of these SNP markers was 847 SNPs/Mb in mei assembly sequences, which was notably lower than that in potato (11,494 SNPs/Mb) [24] and sorghum (2,299 SNPs/Mb) [25]; however, it is similar to that observed in soybean (971 SNPs/Mb) [26]. There was a low level of genetic polymorphism in the two cultivars, in accordance with the perspective that the polymorphisms of SNPs depend on germplasm types, genomic contexts, and mating systems [27]. Most of the nucleotide variants detected were transitions (61.1%), with transversions accounting for 38.9% (Figure 4). The observed transition/transversion (ti/tr) ratio was 1.57, which is consistent with previous reports in potato (1.50) [24] and grape (1.46) [28] but higher than that in soybean (0.92) [26]. The ti/tr ratio appeared to be high when levels of genetic divergence were low and vice versa [29]. The relatively high ti/tr ratio may be indicative of low levels of polymorphism between the two cultivars.

To validate the quality of identified SNPs for a genotyping system, we randomly selected a set of 670 SNPs, which were assembled into an Agilent’s SureSelect solution phase hybridization assay. The 670 SNPs contained 581 SNPs at an average spacing of about 340 Kb widely distributed across eight mei pseudo-chromosomes and 89 SNPs located in assembly sequences which were not anchored to any mei pseudo-chromosome (Figure 2 and Additional file 5). The assay was then applied to 23 mei cultivars and 1 plum cultivar (Table 2).

**Table 2:** List of the cultivars utilized in the dendrogram

**No.**	**Cultivar name**	**Type**	**No.**	**Cultivar name**	**Type**
1	‘Shuangbi Chuizhi’	*P. mume*	13	‘Dayun Zhaoshui’	*P. mume*
2	‘Zao Yudie’	*P. mume*	14	‘Jiang Mei’	*P. mume*
3	‘Xiaohong Changxu’	*P. mume*	15	‘Huang Jinhe’	*P. mume*
4	‘Dayu Zhaoshui’	*P. mume*	16	‘Yudie Longyou’	*P. mume*
5	‘Fenyun Jiangmei’	*P. mume*	17	‘Feng Hou’	*P. mume*
6	‘Yi Nv’	*P. mume*	18	‘Nanjing Hong’	*P. mume*
7	‘Xiao Lve’	*P. mume*	19	‘Xiao Yudie’	*P. mume*
8	‘Nanjing Fuhuangxiang’	*P. mume*	20	‘Danban Lve’	*P. mume*
9	‘Guhong Chuizhi’	*P. mume*	21	‘Jinhong Chuizhi’	*P. mume*
10	‘Hongyan Gongfen’	*P. mume*	22	‘Danban Zhusha’	*P. mume*
11	‘Taohong Zhusha’	*P. mume*	23	‘Fuban Tiaozhi’	*P. mume*
12	‘Dan Fenghou’	*P. mume*	24	‘Ao Li’	*P. salicina*

Captured DNA was sequenced on an Illumina GA II instrument, generating 4.2 G sequencing data with 78 bp reads from the 24 libraries that had been prepared with the SureSelect method (NCBI database under accession SRA063161), and 3.4 G reads passed through the Illumina chastity filter to produce automatic allele calling for each locus. Each library was sequenced to a specific depth, providing a mean ~20-fold mapped coverage of the targeted region. Of 670 SNPs, 89.4% (599 in total) produced non-ambiguous data containing 513 SNPs distributed across eight mei pseudo-chromosomes and 86 SNPs located in assembly sequences that were not anchored to mei pseudo-chromosomes (Figure 2 and Additional file 6). About 85.6% (513 in total) of the 599 SNPs were distributed across the mei pseudo-chromosomes with an average of 64 SNPs per pseudo-chromosome, ranging from a maximum of 117 on pseudo-chromosome 2 to a minimum of 38 on pseudo-chromosome 8 (Figure 2 and Additional file 6).

Polymorphic levels of the 599 SNP loci were estimated using 23 mei cultivars and 1 plum cultivar (Additional file 6). Polymorphism information content (PIC) values ranged between 0.26 and 0.50 (mean 0.45), with 541 of the markers producing PIC values > 0.4, a level which was suitable for biodiversity analyses. Generally, diversity values [expected heterozygosity (H_e_)] for SNPs are low [30]. This is ascribed to their bi-allelic nature. In mei, the observed heterozygosity (H_o_) and H_e_ per locus varied from 0.09 to 0.77 (mean 0.47) and from 0.26 to 0.51 (mean 0.46), respectively (Additional file 6). The mean diversity value (0.46) was higher than the mean values reported for grape (0.30) [28]. However, mei SNPs showed lower diversity values than SSR (0.68) markers [31]. This is a potential drawback of SNPs, but it can be overcome by using a large numbers of markers.

**Figure 5:**
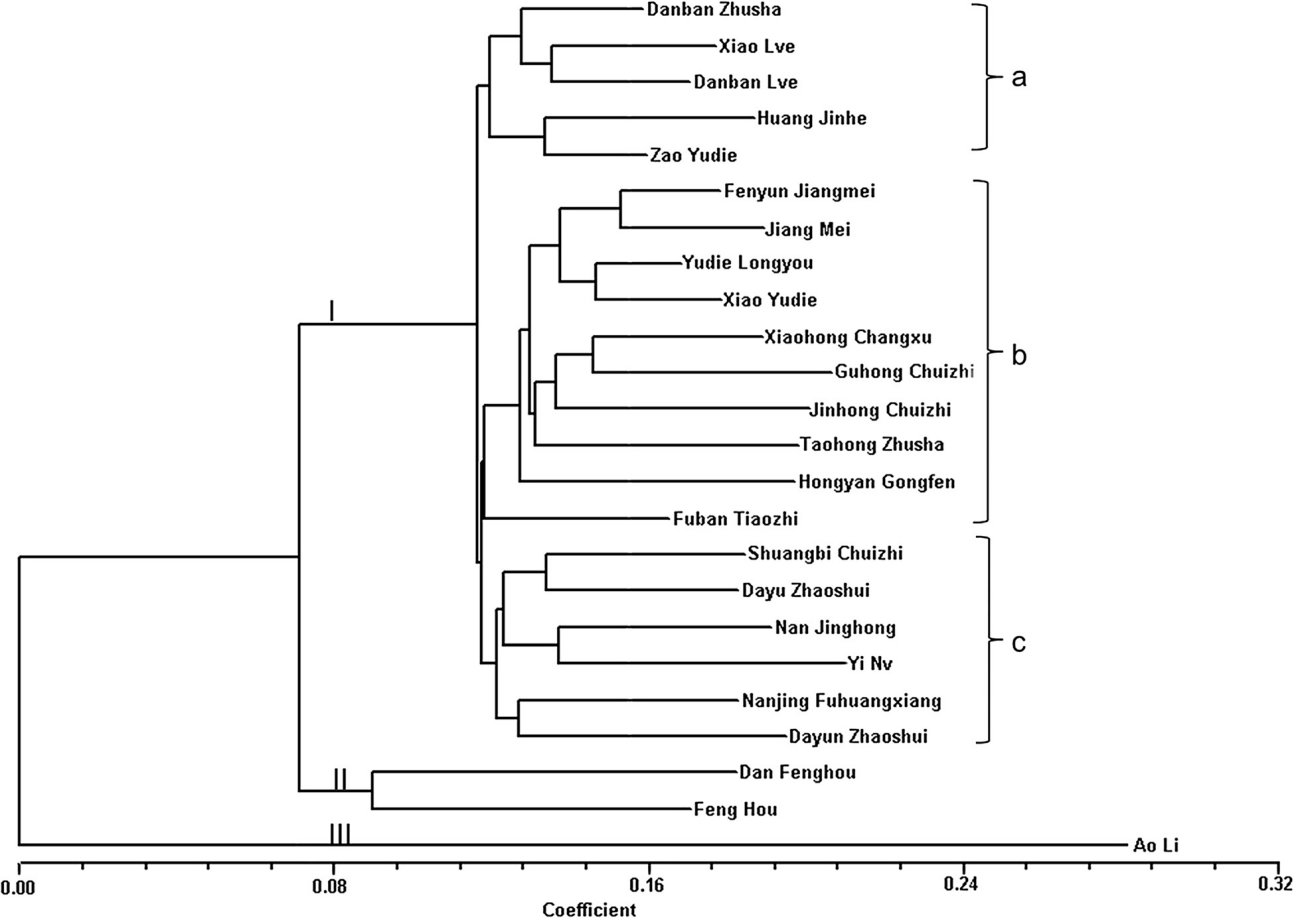
**Phylogeny of 23 cultivars of mei and 1 cultivar of plum.** The dendrogram is constructed using allele callings at 599 SNP loci. All the cultivars were divided into three groups. Groups I–III are the True Mume Branch which contains three subgroups **(a-c)**, Apricot Mei Branch, and plum, respectively.

These SNPs were used to construct a dendrogram for the diverse cultivars of mei and one genotype of plum. The results showed the presence of three major clades (Figure 5). Major clade Ι contained the True Mume Branch (*P. mume*), which is believed to have evolved exclusively from mei without the introgression of foreign genes [1]. Although there were three subgroups (a-c) in the True Mume Branch, most of the cultivars in the subgroups with similar traits did not form groups. Only ‘Jiangmei’ and ‘Fenyun Jiangmei’ of similar traits were grouped together; the same is true for ‘Xiao Lve’ and ‘Danban Lve’. Traits such as plant type, flower type, and flower color are used to differentiate mei cultivars in production [1]. Results demonstrated that mei cultivars possessed a similar genetic pedigree and this conclusion was consistent with those of previous studies [32]. Clade II included the Apricot Mei Branch (*P. mume* var. bungo) consisting of the hybrids of mei and apricot [1]. Our results confirmed the findings of previous studies regarding the hybrid nature of ‘Dan Fenghou’ and ‘Fen Hou’ using random amplified polymorphic DNA (RAPD) and amplified fragment length polymorphism (AFLP) markers [3,33]. Clade III was found to include plum, indicating a relatively distant interspecies relationship between plum and mei. This was consistent with the findings reported in other studies. Internal transcribed spacer (ITS) sequences and EST-SSR markers demonstrated that mei is differentiated from plum species [4,34]. Together, these mei SNP markers were found to be useful in the appraisal of genetic relationships among diverse cultivars of mei and plum.

### InDels as DNA markers

So far, a massive number of InDels have been generated using the NGS platform. These markers ascribed to their high polymorphisms and distribution throughout the genome have been applied to high-resolution genetic mapping, association studies, and map-based cloning [10,12,35]. However, the usefulness of InDels has not been explored in mei genetic and genomic research.

**Figure 6:**
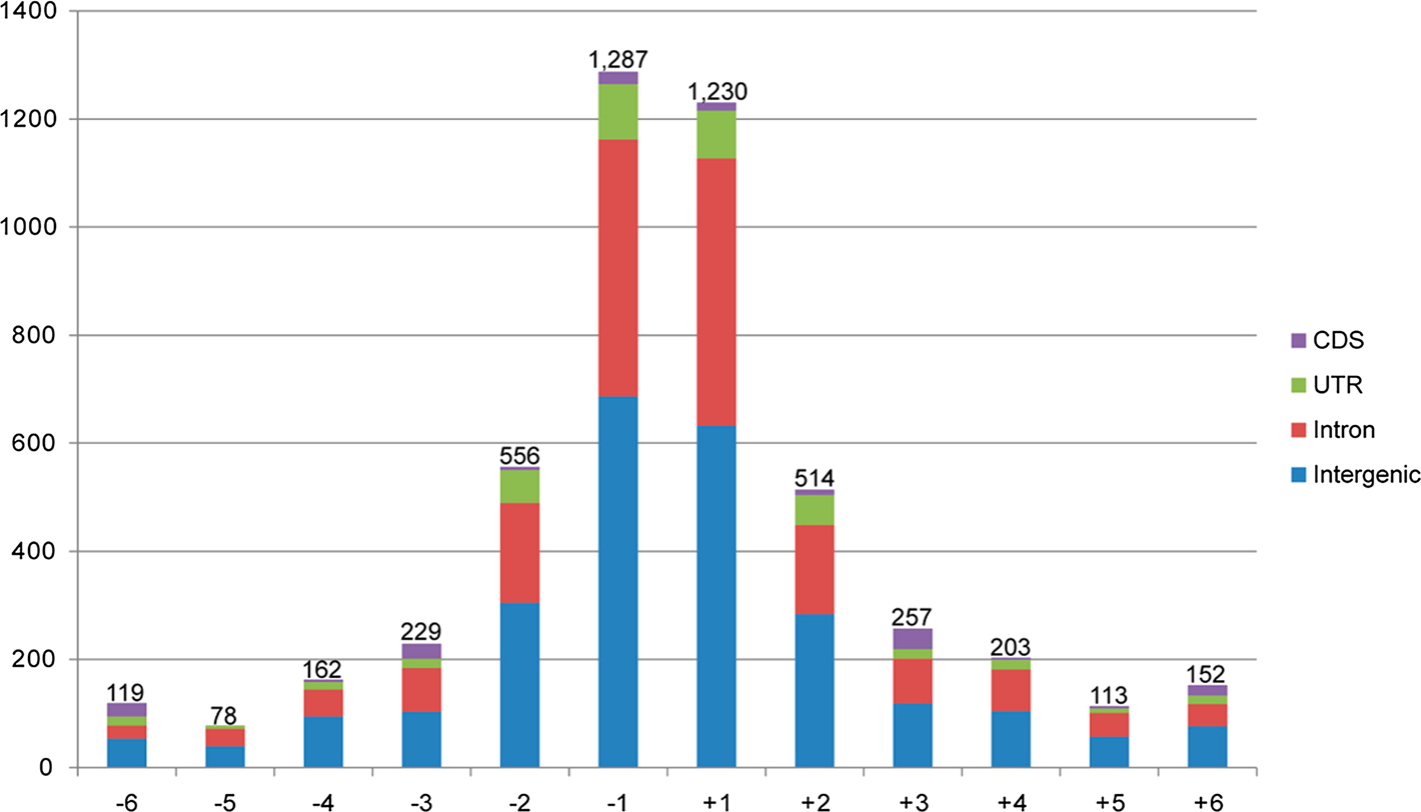
**Distribution of the length of InDels in mei genome.** The x-axis indicates the number of nucleotides of insertions (+) and deletions (−). The y-axis indicates the number of InDels at each length in the CDS, UTR, intron, and intergenic regions.

Whole-genome sequencing can also be used to detect InDel polymorphisms. A total of 4,900 InDels (1–6 bp) including 2,469 insertions and 2,431 deletions were observed in ‘Fenban’ and ‘Kouzi Yudie’ (Additional file 2). They occurred at a frequency of 21 InDels/Mb in mei assembly sequences. The frequency of different types of InDels varied, showing a negative correlation to the number of nucleotides. Mononucleotide InDels (2,517, 51.4%) were the most common type of InDels in genomic regions, following by di- (1,070, 21.8%) and trinucleotide InDels (486, 9.9%), as seen in Figure 6. Most of the InDels in the CDS regions were tri- or hexanucleotides, which could not have been caused by frame shifts as indicated by the similar results detected in the rice, human, and mouse genomes [7,36]. However, mononucleotides were always the most common nucleotides in intergenic regions (Figure 6 and Additional file 2). Out of the total, 2,557 InDels were identified in intergenic regions and 1748, 421, and 174 of these were distributed in introns, untranslated regions (UTR), and CDS, respectively. Despite the minimally abundant distribution within critical sites, such as the CDS and UTR regions (12.1% of total InDels), these InDels can alter mei phenotypes through a variety of mechanisms.

To verify that these InDels were suitable for use as new DNA markers, they were used to successfully design PCR primers (Additional file 2). Twenty pairs of the InDel primers labeled with fluorescent dyes were selected for a survey of polymorphisms among *P. mume* ‘Fenban’ and *P. mume* ‘Kouzi Yudie,’ and five randomly chosen segregating progeny from a cross between the two cultivars (Additional file 2). The PCR analysis indicated that three of the 20 primer pairs produced no products and that there were no polymorphisms among the mapping parents for the two of the 20 primer pairs. Fifteen primers, which gave reliable and stable amplifications and showed large numbers of polymorphisms, were found suitable for use in the construction of a genetic linkage map in the mapping population. However, a detailed analysis of these polymorphic InDels revealed that three showed longer insertions or deletions than expected (Additional file 7). Krawitz et al. demonstrated that a short sequence read including an InDel might be aligned with mismatched bases instead of gaps [37]. They accomplished this using a BWA short-read mapping tool, which generated a high rate of variant bases at InDel positions [37]. Thus, the mismatched InDels observed in our study may be attributed to alignment with mismatched bases instead of gaps. As a result, the predicted InDel lengths were shorter than those observed by successful PCR amplifications of fragments containing InDels. The high ratio of successful InDel amplifications showed that the detected InDel markers may be suitable for use in the construction of genetic linkage maps.

### SSRs as DNA markers

The SSRs were also detected in the sequences common to both ‘Fenban’ and ‘Kouzi Yudie’ in a sequencing dataset mapped to the mei reference genome. We identified 7,063 putative polymorphic SSRs between the two cultivars. Mononucleotide repeats were the most common, with 3,083 (43.7%) found. They were followed by 2,835 dinucleotide repeats (40.1%) and 837 trinucleotide repeats (11.8%) (Table 3). The frequency of SSRs decreased as the repeat motifs increased in length. This was consistent with previous studies in rice [38] and Brachypodium [39]. The formation of SSRs can be attributed to the major mechanism, the spontaneous creation of proto-microsatellites from unique sequences by substitutions and insertions [40] followed by elongations and expansions of these proto-microsatellites by transposable elements [41]. We speculate that the proto-microsatellites are more likely to include short motifs than long motifs. This could explain why mononucleotides were the most abundant SSRs and why penta- and hexanucleotides were rare.

**Table 3:** Distribution of 7,063 putative polymorphic SSRs identified between ‘Fenban’ and ‘Kouzi Yudie’

**Motif**	**Counts**	**%**	**Average motif length**	**Number of repeats**									
				**4**	**5**	**6**	**7**	**8**	**9**	**10**	**>10**	**Class I**	**Class II**
Mononucleotide	3,083	43.7%	15								3,083	346	2,737
Dinucleotide	2,835	40.1%	21			556	345	327	261	241	1,105	1,346	1,489
Trinucleotide	837	11.8%	16	358	214	134	64	33	16	5	13	131	706
Tetranucleotide	206	2.9%	19	122	55	21	1	4	2	1	0	122	84
Pentanucleotide	58	0.9%	22	43	13	1	1	0	0	0	0	58	0
Hexanucleotide	44	0.6%	26	32	9	3	0	0	0	0	0	44	0
Total	7,063	100.0%	20	555	291	715	411	364	279	247	4,201	2,047	5,016

SSR loci have been categorized into two classes based on the lengths of SSR repeat motifs: hypervariable class I SSRs (≥ 20 bp) and potentially variable class II SSRs (≥ 12 bp and < 20 bp) [11]. Among the polymorphic SSRs in the two cultivars, class II SSRs (5,016) were significantly more common than the class I SSRs (2,047) (Table 3). Similar patterns have been observed in rice [38] and papaya [42]. These results can be attributed to the fact that class II SSRs are composed of short repeats, which are more tolerant to mutations than class I SSRs [42]. However, class I SSRs are more polymorphic than class II SSRs, as demonstrated by the experimental data reported for rice [38], Brachypodium [39], and papaya [42]. Class II SSRs tend to be less variable because of their smaller chance of slipped-strand mispairing over the expansion of shorter SSR motifs than longer motifs [11]. On the basis of SSR motif length, the dinucleotide repeats (1,346) were the most common motifs in class I SSRs, as indicated by the reports from the five plant species analyzed by Mun et al. [43]. Mononucleotides were the most abundant in class II SSRs, which may be explained by the fact that polymerase slippage rates are higher in dinucleotides than in other repeat motifs. These results are in accordance with the data from human [44] and fruit fly SSRs [45].

Polymorphic SSRs with different repeat motifs were also found in the two cultivars. The most common di- and trinucleotide motifs were AG/CT (55.8%) and AAT/ATT (35.5%); however, CG/CG was not observed in either cultivar and CCG/CGG (0.6%) was rare (Additional file 8). AT-rich polymorphic repeat motifs of SSRs were more common than GC-rich repeat motifs in the mapping parents, as indicated in previous reports from eggplant [8] and papaya [42]. According to previous studies, the (CTG)_n_, (CCG)_n_, (AT)_n_, and (GC)_n_, all of which have hairpin structures and self-complementary repeat motifs, accumulate readily in the mei genome [46,47]. However, methylated cytosine can mutate to thymine easily, which may explain the scarcity of GC-rich repeats [48].

All of these polymorphic SSRs were used to design PCR primers (Additional file 3). In order to assess the SSR polymorphisms among the parental lines and five segregating progeny, twenty pairs of SSR primers were designed and labeled with fluorescent dyes. Eighteen pairs of 20 primers were used for the successful amplification, of which fifteen pairs were suitable for constructing the genetic map between the two cultivars (Additional file 9). A few SSR primers could not be used for successful amplification as indicated by null alleles, which may have been generated by some mutations involving substitutions within primer binding sites and SSR deletions [49]. However, the bulk of the primers could amplify the SSRs successfully, demonstrating the large number of polymorphisms. These observations provide insight into the use of SSRs for the construction of high-resolution genetic maps of mei cultivars in the near future.

## Conclusion

In this study, we observed a large number of putative polymorphic SNPs, InDels, and SSRs between ‘Fenban’ and ‘Kouzi Yudie’ using low-depth whole genome sequencing, which present a new methodology and extensive data. These putative polymorphic markers could facilitate the construction of high-density genetic linkage maps, and accelerate QTL analyses, GWAS, genomic selection, and MAS breeding programs in mei.

## Methods

### Plant materials and DNA extraction

Twenty-three mei cultivars from the mei germplasm bank in the China Mei Flower Research Center (Wuhan city, China) and one plum cultivar from the Beijing Botanical Garden (Beijing city, China) were collected to perform sequence capture using Agilent’s SureSelect solution phase hybridization assay (Table 2). All DNA samples were extracted from young leaves using the plant genomic DNA extraction Kit (TIANGEN, Beijing, China) following the manufacturer’s protocol.

### Sequence mapping and SNP calling

The genome sequences for *P. mume* ‘Fenban’ and *P. mume* ‘Kouzi Yudie’ were downloaded from NCBI database under accession SRA057102. All sequences were aligned to the mei reference genome (http://prunusmumegenome.bjfu.edu.cn./) using BWA software (ver. 0.5.1) [15] with the cutoff maximum of three mismatches in 90 bp and 2 mismatches in 45 bp. We excluded reads that could be mapped to different genomic positions so as to detect high-quality DNA polymorphic markers.

Uniquely mapped pair-end results were used to perform SNP calling using SOAPsnp [50]. Subsequently, the SNPs with overall sequencing depths of more than 8, quality scores over 30, and at least 4 uniquely mapped reads per allele were extracted.

### InDels detection

To detect InDels in uniquely mapped sequences, another mapping process was performed, allowing a gap using BWA software (ver. 0.5.1) [15]. InDels (1–6 bp) were then called using SOAPindel as described in a previous study [17]. Each InDel locus contained an InDel motif and two unique flanking sequences of less than 195 bp on each side of that motif. The InDels were classified as putative polymorphisms if the lengths of the InDel motifs from the two cultivars varied by least 1 bp.

### SSRs identification

Uniquely mapped reads were used to detect SSRs using the computer program MISA (MIcroSAtellites identification tool, http://pgrc.ipk-gatersleben.de/misa). Minimum repeat lengths for SSR findings were set as 12 bp for mono- to trinucleotides, 16 bp for tetranucleotides, 20 bp for pentanucleotides, 24 bp for hexanucleotides. An SSR locus contained a repeat motif and two unique flanking sequences of 180 bp on each side of the repeat motif. On the basis of these sizes, the SSRs were classified as polymorphisms if the lengths of repeat motifs from the two cultivars varied at least by 2 bp.

### Annotation of SNPs, InDels and SSRs

The positions of SNPs, InDels and SSRs were identified as CDS, intron, 5′UTR, 3′UTR and intergenic regions according to mei genome GFF files, and each CDS containing these markers were assigned to one or more function annotations using mei annotation project files. These files were downloaded from the Mei Genome Database (http://prunusmumegenome.bjfu.edu.cn). The annotated sequences were then mapped to high level categories using these mei annotation project files according to the three main GO categories (biological process, molecular function, and cellular component). SNPs in the CDS regions were divided into synonymous and non-synonymous amino acid substitutions.

### Chip design

Using the SureSelect method from Agilent [51], a total of 670 biotinylated RNA probes, each 120 nucleotides in length (Additional file 5), were designed to capture the desired DNA fragments from a pool of 24 genotype DNA fragments. The proportions of the targeted intron, CDS, UTR, and intergenic sequences were 17.5%, 25.5%, 4.8%, and 52.2%, respectively. Capture assay was hybridized with 24 genotypes from genomic libraries labeled with different barcodes. Captured DNA was then sequenced on the Illumina GAII instrument, generating 4.2 G 78 bp reads.

### Chip capture library preparation, hybridization and sequencing

At least 3 μg of genomic DNA of each of the 24 accessions was placed in 80 μl TE-buffer and fragmented using the Covaris instrument. This was followed by end repair, A-tailing, and BGI PE index adapter ligation, as described in the Illumina DNA library preparation protocol [52].

Adapter ligated DNA was run on a 2% TAE agarose gel, and the region of the gel with fragments in the range of 200–250 bp was excised. The DNA was purified using a gel extraction kit (Qiagen) and eluted in 90 μl EB. The adapter ligated and size-selected DNA was amplified in 50 μl PCR. The PCR reaction contained 3 μl of DNA, 18 ml H_2_O, 2 μl primer 1.1 (Illumina), 2 μl primer 2.1 (Illumina), and 25 μl Phusion master mix (Finnzymes). PCR amplification conditions were as follows: 2 min at 95°C; 4 cycles of 15 s at 95 °C, 30 s at 60°C, and 30 s at 72°C; then 5 min at 72°C. The reaction product was purified using a QIAquick PCR purification kit (Qiagen) and eluted into 20 μl EB.

SureSelect solution phase hybridization was conducted according to the manufacturer’s (Agilent) standard protocol. The buffers #1, #2, #3, and #4 from the SureSelect kit were mixed to prepare the hybridization solution, which was incubated at 65°C. In parallel, the 300 ng of each DNA library were pooled with the blocker #1, #2, and #3 reagents (Agilent), denatured for 5 min at 95°C, and then incubated at 65°C in a thermal cycler (MJ Research). We then mixed 12 μl of hybridization solution, 5 μl of mixed SureSelect Oligo Capture Library, 11 μl of the DNA library, 1 μl H_2_O, and 1 μl RNase block (Agilent), incubated for 24 hours at 65°C in a thermal cycler (MJ Research) and captured with the Streptavidin M-280 Dynabeads (Invitrogen). The reaction product was then purified with the MinElute PCR purification kit (Qiagen) according to the manufacturer’s protocol. The purified DNA was enriched by 50 μl PCR reactions containing 15 μl of elution production, 8 μl H_2_O, 1 μl primer 1.1 (Illumina), 1 μl primer 2.1 (Illumina), and 25 μl Phusion master mix (Finnzymes). The PCR conditions were performed as described above. The PCR products were pooled and purified with Ampure beads (Beckman) and eluted using 50 μl EB. The quality of the capture sample was assessed using a Qubit® dsDNA HS Assay Kit (Invitrogen) prior to its sequencing on Illumina GAII instrument as PE 78 bp reads.

### Assessment of genetic diversity as indicated by SureSelect hybrid capture system

Agilent SureSelect liquid-based hybrid capture arrays were used for SNPs genotyping. The allele calling for each locus was identified using SOAPsnp [50]. Sites meeting the following criteria were identified: overall sequencing depth of over 15; quality score over 30; at least 4 uniquely mapped reads per allele. These sites were referred to as high-confidence calls in our study. For each SNP locus, the number of alleles (N_a_), H_o_, and H_e_ was calculated using GenePop version 4.0 [53]. The PIC was calculated using the following formula: *PIC* = 1-∑*P*_*i*_^*2*^, where *P*_*i*_ is the *i*th SNP allele frequency [54]. Each SNP locus was scored for the presence (1) or absence (0) of genotype. The data set was used to compile a binary matrix describing 24 cultivar genotypes based on 599 polymorphic co-dominant SNP markers. The genetic similarity coefficient among the genotypes was estimated using NTSYS-pc software (version 2.10) [55]. A dendrogram was generated for the analysis of genetic diversity among mei and plum genotypes based on Neighbor-joining (NJ) method.

### SSR and InDel primers design and experimental validation

The putative polymorphic SSR and InDel loci were scanned using Primer 3 (v. 1.1.4) to design oligonucleotide primers flanking the repeats [56]. The optimized input parameters were as follows: product size: 100–300 bp; primer size: 18–25 bp; primer Tm: 50-60°C; primer GC content: 40-60%.

Of these putative polymorphic SSRs and InDels, we randomly chose 20 primer pairs labeled with fluorescent dyes and amplified among the parental lines and five segregating progeny, respectively. The total genomic DNA from their fresh young leaves was extracted as described above. The SSR and InDel genotypes were performed using a primer strategy, including a forward primer labeled with FAM (Beijing Microread Genetics Co., Ltd, Beijing, China), and a regular reverse primer. The PCR reactions of SSRs and InDels were respectively conducted in a 10 μl mixture. The same mixtures included 50 ng of the genomic DNA, 1 μl of 10 × buffer [20 mM Tris–HCl (pH 8.4), 20 mM KCl, 10 mM (NH_4_)_2_SO_4_, and 1.5 mM MgCl_2_], 1.2 μl of 2.5 mM dNTP, and 0.6 U of Taq DNA polymerase (Promega, Madison, WI, USA). The different mixtures were as follows: 0.9 μl of 10 uM each of forward and reverse primers for SSRs, and 1 μl of these for InDels and added ddH_2_O to the total volume. The PCR amplifications of SSRs and InDels were performed with the following program: 5 min at 95°C; followed by 25 cycles of 40 s at 95°C, 30 s at the optimized annealing temperature for each primers (Additional files 2 and 3), 40 s at 72°C, and then a final step for 5 min at 72°C. The PCR products of SSRs and InDels were resolved on an ABI 3730 fluorescent analyzer (Applied Biosystems, Foster City, CA, USA) with the ROX 400 HD as size standard. Data were then analyzed using GeneMapper version 3.7 software (Applied Biosystems, Foster City, CA, USA).

## Abbreviations

SNPs: Single nucleotide polymorphisms; InDels: Insertions-deletions; SSRs: Simple sequence repeats; QTL: Quantitative trait locus; MAS: Marker-associated selection; GWAS: Genome-wide association study; LD: Linkage disequilibrium; NGS: Next-generation sequencing; RAD: Restriction-site-associated DNA; NJ: Neighbor-joining; GA: Genome Analyzer; BWA: Burrows-Wheeler alignment tool; CDS: Coding sequences; GO: Gene ontology; PIC: Polymorphism information content; He: Expected heterozygosity; Ho: Observed heterozygosity; Na: Numbers of alleles; RAPD: Random amplified polymorphic DNA; AFLP: Amplified fragment length polymorphism; ITS: Internal transcribed spacer.

## Competing interests

The authors declare that they have no competing interests.

## Authors’ contributions

SL performed bioinformatics and manuscript draft writing. ZQ designed the study and revised this manuscript. SL, XZ, GY and LJ carried out the experiments. YW, CT, CM, and PH participated in the design and coordination the study. All authors read and approved the final manuscript.

## Supplementary Material

Additional file 1 Detailed information of 200,627 polymorphic SNPs identified between ‘Fenban’ and ‘Kouzi Yudie’. Click here for file

Additional file 2 Detailed information of 4,900 polymorphic InDels detected between ‘Fenban’ and ‘Kouzi Yudie’. Click here for file

Additional file 3 Detailed information of 7,063 polymorphic SSRs found between ‘Fenban’ and ‘Kouzi Yudie’. Click here for file

Additional file 4 Functional annotation of 9,557 genes containing polymorphic markers in mei. Click here for file

Additional file 5 Characteristics of 670 polymorphic SNP probe loci developed in ‘Fenban’ and ‘Kouzi Yudie’. Click here for file

Additional file 6 Polymorphisms of 599 SNP markers based on 23 mei genotypes and 1 plum genotype. Click here for file

Additional file 7 **Amplifications of polymorphic InDel primers labeled by FAM fluorescent dyes indicated the long InDels compared with the expected sizes.** Panels indicated data from ‘Fenban’ (FB) and ‘Kouzi Yudie’ (KZYD) and their F_1_ hybrids (HB): (A) and (B) loci heterozygosity in the ‘Fenban’, two alleles; (C) loci heterozygosity in the ‘Kouzi Yudie’, two alleles. Click here for file

Additional file 8 Relative frequency for mono-, di-, and trinucleotides of SSR repeat motifs. Click here for file

Additional file 9 **Examples of amplifications of SSR primers labeled with FAM fluorescent dyes.** Panels indicated data from ‘Fenban’ (FB) and ‘Kouzi Yudie’ (KZYD) and their F_1_ hybrids (HB): (A) locus heterozygosities in the ‘Fenban’, two alleles; (B) locus heterozygosities in the ‘Kouzi Yudie’, two alleles; (C) locus heterozygosities in parental lines, two alleles; (D) locus heterozygosities in parental lines, three alleles; (E) locus heterozygosities in parental lines, four alleles; (F) locus homozygosity in parental lines. Click here for file

## References

[B1] Chen JY Chinese Mei Flowers (in Chinese) 1996 Haikou, China: Hainan Publishing House

[B2] Chu MY China Fruit Records - Mei (in Chinese) 1999 Beijing: China Forestry Press

[B3] Yang C-D Zhang J-W Yan X-L Bao M-Z Genetic relatedness and genetic diversity of ornamental mei (*Prunus mume* Sieb. et Zucc.) as analysed by AFLP markers Tree Genetics & Genomes 2008 4 2 255 262 10.1007/s11295-007-0106-024099088

[B4] Li X Shangguan L Song C Wang C Gao Z Yu H Fang J Analysis of expressed sequence tags from *Prunus mume* flower and fruit and development of simple sequence repeat markers BMC genetics 2010 11 66 20626882 10.1186/1471-2156-11-66 PMC2920227

[B5] Zhang Q Chen W Sun L Zhao F Huang B Yang W Tao Y Wang J Yuan Z Fan G The genome of *Prunus mume* Nat Commun 2012 3 1318 23271652 10.1038/ncomms2290 PMC3535359

[B6] Sun L Yang W Zhang Q Cheng T Pan H Xu Z Zhang J Chen C Genome-wide characterization and linkage mapping of simple sequence repeats in mei (*Prunus mume* Sieb. et Zucc.) PloS one 2013 8 3 e59562 10.1371/journal.pone.0059562 23555708 PMC3610739

[B7] Arai-Kichise Y Shiwa Y Nagasaki H Ebana K Yoshikawa H Yano M Wakasa K Discovery of genome-wide DNA polymorphisms in a landrace cultivar of Japonica rice by whole-genome sequencing Plant Cell Physiol 2011 52 2 274 282 10.1093/pcp/pcr003 21258067 PMC3037082

[B8] Barchi L Lanteri S Portis E Acquadro A Vale G Toppino L Rotino GL Identification of SNP and SSR markers in eggplant using RAD tag sequencing BMC Genomics 2011 12 304 10.1186/1471-2164-12-304 21663628 PMC3128069

[B9] Ren Y Zhao H Kou Q Jiang J Guo S Zhang H Hou W Zou X Sun H Gong G A high resolution genetic map anchoring scaffolds of the sequenced watermelon genome PloS One 2012 7 1 e29453 10.1371/journal.pone.0029453 22247776 PMC3256148

[B10] Wang Y Sun S Liu B Wang H Deng J Liao Y Wang Q Cheng F Wang X Wu J A sequence-based genetic linkage map as a reference for *Brassica rapa* pseudochromosome assembly BMC Genomics 2011 12 239 10.1186/1471-2164-12-239 21569561 PMC3224973

[B11] Temnykh S DeClerck G Lukashova A Lipovich L Cartinhour S McCouch S Computational and experimental analysis of microsatellites in rice (*Oryza sativa* L.): frequency, length variation, transposon associations, and genetic marker potential Genome Res 2001 11 8 1441 1452 10.1101/gr.184001 11483586 PMC311097

[B12] Mills RE Luttig CT Larkins CE Beauchamp A Tsui C Pittard WS Devine SE An initial map of insertion and deletion (INDEL) variation in the human genome Genome Res 2006 16 9 1182 1190 10.1101/gr.4565806 16902084 PMC1557762

[B13] Kump KL Bradbury PJ Wisser RJ Buckler ES Belcher AR Oropeza-Rosas MA Zwonitzer JC Kresovich S McMullen MD Ware D Genome-wide association study of quantitative resistance to southern leaf blight in the maize nested association mapping population Nat Genet 2011 43 2 163 168 10.1038/ng.747 21217757

[B14] Resende MD Resende MF Jr Sansaloni CP Petroli CD Missiaggia AA Aguiar AM Abad JM Takahashi EK Rosado AM Faria DA Genomic selection for growth and wood quality in Eucalyptus: capturing the missing heritability and accelerating breeding for complex traits in forest trees New Phytol 2012 194 1 116 128 10.1111/j.1469-8137.2011.04038.x 22309312

[B15] Li H Durbin R Fast and accurate long-read alignment with Burrows-Wheeler transform Bioinformatics 2010 26 5 589 595 10.1093/bioinformatics/btp698 20080505 PMC2828108

[B16] Jander G Norris SR Rounsley SD Bush DF Levin IM Last RL Arabidopsis map-based cloning in the post-genome era Plant Physiol 2002 129 2 440 450 10.1104/pp.003533 12068090 PMC1540230

[B17] Xu X Liu X Ge S Jensen JD Hu F Li X Dong Y Gutenkunst RN Fang L Huang L Resequencing 50 accessions of cultivated and wild rice yields markers for identifying agronomically important genes Nat Biotechnol 2012 30 1 105 111 22158310 10.1038/nbt.2050

[B18] Lai J Li R Xu X Jin W Xu M Zhao H Xiang Z Song W Ying K Zhang M Genome-wide patterns of genetic variation among elite maize inbred lines Nat Genet 2010 42 11 1027 1030 10.1038/ng.684 20972441

[B19] Morgante M Hanafey M Powell W Microsatellites are preferentially associated with nonrepetitive DNA in plant genomes Nat Genet 2002 30 2 194 200 10.1038/ng822 11799393

[B20] Vali U Brandstrom M Johansson M Ellegren H Insertion-deletion polymorphisms (indels) as genetic markers in natural populations BMC Genet 2008 9 8 18211670 10.1186/1471-2156-9-8 PMC2266919

[B21] Mills RE Pittard WS Mullaney JM Farooq U Creasy TH Mahurkar AA Kemeza DM Strassler DS Ponting CP Webber C Natural genetic variation caused by small insertions and deletions in the human genome Genome Res 2011 21 6 830 839 10.1101/gr.115907.110 21460062 PMC3106316

[B22] Clark RM Schweikert G Toomajian C Ossowski S Zeller G Shinn P Warthmann N Hu TT Fu G Hinds DA Common sequence polymorphisms shaping genetic diversity in *Arabidopsis thaliana* Science 2007 317 5836 338 342 10.1126/science.1138632 17641193

[B23] Lam HM Xu X Liu X Chen W Yang G Wong FL Li MW He W Qin N Wang B Resequencing of 31 wild and cultivated soybean genomes identifies patterns of genetic diversity and selection Nat Genet 2010 42 12 1053 1059 10.1038/ng.715 21076406

[B24] Simko I Haynes KG Jones RW Assessment of linkage disequilibrium in potato genome with single nucleotide polymorphism markers Genetics 2006 173 4 2237 2245 10.1534/genetics.106.060905 16783002 PMC1569688

[B25] Hamblin MT Mitchell SE White GM Gallego J Kukatla R Wing RA Paterson AH Kresovich S Comparative population genetics of the panicoid grasses: sequence polymorphism, linkage disequilibrium and selection in a diverse sample of *sorghum bicolor* Genetics 2004 167 1 471 483 10.1534/genetics.167.1.471 15166170 PMC1470838

[B26] Zhu YL Song QJ Hyten DL Van Tassell CP Matukumalli LK Grimm DR Hyatt SM Fickus EW Young ND Cregan PB Single-nucleotide polymorphisms in soybean Genetics 2003 163 3 1123 1134 12663549 10.1093/genetics/163.3.1123 PMC1462490

[B27] Schneider K Kulosa D Soerensen T Möhring S Heine M Durstewitz G Polley A Weber E Jamsari L Lein J Analysis of DNA polymorphisms in sugar beet (*Beta vulgaris* L.) and development of an SNP-based map of expressed genes Theor Appl Genet 2007 115 5 601 615 10.1007/s00122-007-0591-4 17622508

[B28] Lijavetzky D Cabezas JA Ibanez A Rodriguez V Martinez-Zapater JM High throughput SNP discovery and genotyping in grapevine (*Vitis vinifera* L.) by combining a re-sequencing approach and SNPlex technology BMC genomics 2007 8 424 10.1186/1471-2164-8-424 18021442 PMC2212664

[B29] Yang Z Yoder AD Estimation of the transition/transversion rate bias and species sampling J Mol Evol 1999 48 3 274 283 10.1007/PL00006470 10093216

[B30] Weir B Genetic data analysis II 1996 Sunderland, MA: Sinauer Associates, Inc

[B31] Hayashi K Shimazu K Yaegaki H Yamaguchi M Iketani H Yamamoto T Genetic diversity in fruiting and flower-ornamental Japanese apricot (*Prunus mume*) germplasms assessed by SSR markers Breed Sci 2008 58 401 410 10.1270/jsbbs.58.401

[B32] Li X Wang Y Wang B Wang C Shangguan L HUANG Z Fang J Genetic relationships between fruiting and flowering mei (*Prunus mume*) cultivars using SNP markers Journal of Horticultural Science & Biotechnology 2010 85 4 329 33424099392

[B33] Shimada T Haji T Yamaguchi M Takeda T Nomura K Yoshida M Classification of mume (*Prunus mume* Sieb. et Zucc.) by RAPD assay Journal of the Japanese Society for Horticultural Science 1994 63 3 543 551 10.2503/jjshs.63.543

[B34] Lee S Wen J A phylogenetic analysis of Prunus and the Amygdaloideae (Rosaceae) using ITS sequences of nuclear ribosomal DNA Am J Bot 2001 88 1 150 160 10.2307/2657135 11159135

[B35] Pacurar DI Pacurar ML Street N Bussell JD Pop TI Gutierrez L Bellini C A collection of INDEL markers for map-based cloning in seven Arabidopsis accessions J Exp Bot 2012 63 7 2491 2501 10.1093/jxb/err422 22282537 PMC3346218

[B36] Taylor MS Ponting CP Copley RR Occurrence and consequences of coding sequence insertions and deletions in Mammalian genomes Genome Res 2004 14 4 555 566 10.1101/gr.1977804 15059996 PMC383299

[B37] Krawitz P Rodelsperger C Jager M Jostins L Bauer S Robinson PN Microindel detection in short-read sequence data Bioinformatics 2010 26 6 722 729 10.1093/bioinformatics/btq027 20144947

[B38] Zhang Z Deng Y Tan J Hu S Yu J Xue Q A genome-wide microsatellite polymorphism database for the indica and japonica rice DNA Res 2007 14 1 37 45 10.1093/dnares/dsm005 17452422 PMC2779893

[B39] Sonah H Deshmukh RK Sharma A Singh VP Gupta DK Gacche RN Rana JC Singh NK Sharma TR Genome-wide distribution and organization of microsatellites in plants: an insight into marker development in Brachypodium PloS one 2011 6 6 e21298 10.1371/journal.pone.0021298 21713003 PMC3119692

[B40] Messier W Li SH Stewart CB The birth of microsatellites Nature 1996 381 6582 483 8632820 10.1038/381483a0

[B41] Wilder J Hollocher H Mobile elements and the genesis of microsatellites in dipterans Mol Biol Evol 2001 18 3 384 392 10.1093/oxfordjournals.molbev.a003814 11230539

[B42] Wang J Chen C Na J-K Yu Q Hou S Paull R Moore P Alam M Ming R Genome-wide comparative analyses of microsatellites in papaya Trop Plant Biol 2008 1 3 278 292

[B43] Mun JH Kim DJ Choi HK Gish J Debelle F Mudge J Denny R Endre G Saurat O Dudez AM Distribution of microsatellites in the genome of *Medicago truncatula*: a resource of genetic markers that integrate genetic and physical maps Genetics 2006 172 4 2541 2555 16489220 10.1534/genetics.105.054791 PMC1456377

[B44] Weber JL Informativeness of human (dC-dA)n.(dG-dT)n polymorphisms Genomics 1990 7 4 524 530 10.1016/0888-7543(90)90195-Z 1974878

[B45] Schlotterer C Ritter R Harr B Brem G High mutation rate of a long microsatellite allele in Drosophila melanogaster provides evidence for allele-specific mutation rates Mol Biol Evol 1998 15 10 1269 1274 10.1093/oxfordjournals.molbev.a025855 9787433

[B46] Mitas M Yu A Dill J Kamp TJ Chambers EJ Haworth IS Hairpin properties of single-stranded DNA containing a GC-rich triplet repeat: (CTG)15 Nucleic Acids Res 1995 23 6 1050 1059 10.1093/nar/23.6.1050 7731793 PMC306804

[B47] Gacy AM Goellner G Juranic N Macura S McMurray CT Trinucleotide repeats that expand in human disease form hairpin structures in vitro Cell 1995 81 4 533 540 10.1016/0092-8674(95)90074-8 7758107

[B48] Chan SW Henderson IR Jacobsen SE Gardening the genome: DNA methylation in *Arabidopsis thaliana* Nature reviews Genetics 2005 6 5 351 360 15861207 10.1038/nrg1601

[B49] Varshney RK Graner A Sorrells ME Genic microsatellite markers in plants: features and applications Trends Biotechnol 2005 23 1 48 55 10.1016/j.tibtech.2004.11.005 15629858

[B50] Li R Li Y Fang X Yang H Wang J Kristiansen K SNP detection for massively parallel whole-genome resequencing Genome Res 2009 19 6 1124 1132 10.1101/gr.088013.108 19420381 PMC2694485

[B51] Gnirke A Melnikov A Maguire J Rogov P LeProust EM Brockman W Fennell T Giannoukos G Fisher S Russ C Solution hybrid selection with ultra-long oligonucleotides for massively parallel targeted sequencing Nat Biotechnol 2009 27 2 182 189 10.1038/nbt.1523 19182786 PMC2663421

[B52] Quail MA Kozarewa I Smith F Scally A Stephens PJ Durbin R Swerdlow H Turner DJ A large genome center’s improvements to the Illumina sequencing system Nat Methods 2008 5 12 1005 1010 10.1038/nmeth.1270 19034268 PMC2610436

[B53] Raymond M Rousset F GENEPOP (Version 1.2): Population Genetics Software for Exact Tests and Ecumenicism J Hered 1995 86 3 248 249

[B54] Anderson JA Churchill GA Autrique JE Tanksley SD Sorrells ME Optimizing parental selection for genetic linkage maps Genome 1993 36 1 181 186 10.1139/g93-024 18469981

[B55] Rohlf FJ NTSYS-pc: numerical taxonomy and multivariate analysis system: Applied Biostatistics 1992

[B56] Rozen S Skaletsky H Primer3 on the WWW for general users and for biologist programmers Methods Mol Biol 2000 132 365 386 10547847 10.1385/1-59259-192-2:365

