# Supplementary material for: Genome-wide DNA polymorphisms in two cultivars of mei (Prunus mume sieb. et zucc.)
Source: BMC Genet. 2013 Oct 6;14:98. doi: 10.1186/1471-2156-14-98 (PMC3851432; doi:10.1186/1471-2156-14-98)
Supplement: Additional file 7 — Amplifications of polymorphic InDel primers labeled by FAM fluorescent dyes indicated the long InDels compared with the expected sizes. Panels indicated data from ‘Fenban’ (FB) and ‘Kouzi Yudie’ (KZYD) and their F1 hybrids (HB): (A) and (B) loci heterozygosity in the ‘Fenban’, two alleles; (C) loci heterozygosity in the ‘Kouzi Yudie’, two alleles. [file 1471-2156-14-98-S7.doc]

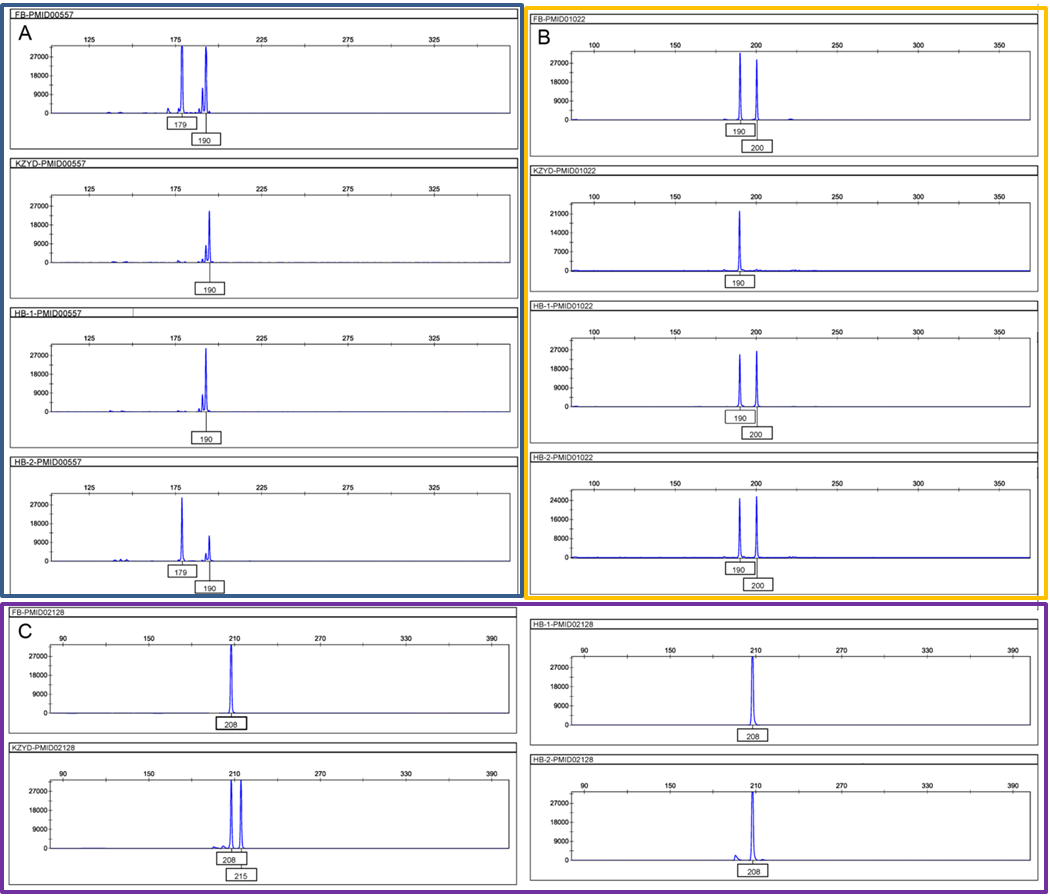


**Additional file 7. Amplifications of polymorphic InDel primers labeled by FAM fluorescent dyes indicated the long InDels compared with the expected sizes.** Panels indicated data from 'Fenban' (FB) and 'Kouzi Yudie' (KZYD) and their F_1_ hybrids (HB): (A) and (B) loci heterozygosity in the 'Fenban', two alleles; (C) loci heterozygosity in the 'Kouzi Yudie', two alleles.
