# Supplementary material for: Genome-wide DNA polymorphisms in two cultivars of mei (Prunus mume sieb. et zucc.)
Source: BMC Genet. 2013 Oct 6;14:98. doi: 10.1186/1471-2156-14-98 (PMC3851432; doi:10.1186/1471-2156-14-98)
Supplement: Additional file 9 — Examples of amplifications of SSR primers labeled with FAM fluorescent dyes. Panels indicated data from ‘Fenban’ (FB) and ‘Kouzi Yudie’ (KZYD) and their F1 hybrids (HB): (A) locus heterozygosities in the ‘Fenban’, two alleles; (B) locus heterozygosities in the ‘Kouzi Yudie’, two alleles; (C) locus heterozygosities in parental lines, two alleles; (D) locus heterozygosities in parental lines, three alleles; (E) locus heterozygosities in parental lines, four alleles; (F) locus homozygosity in parental lines. [file 1471-2156-14-98-S9.doc]

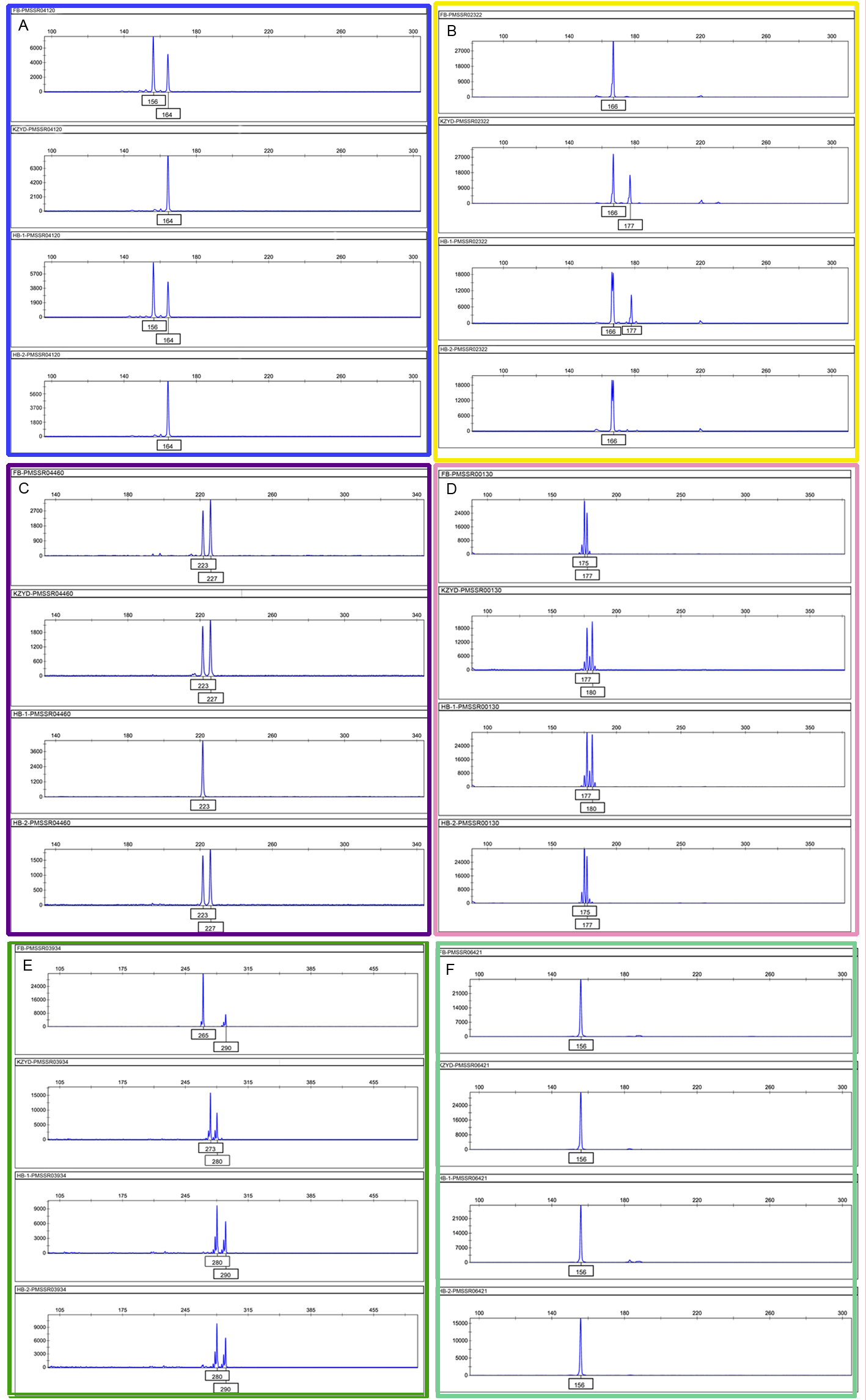


**Additional file 9. Examples of amplifications of SSR primers labeled with FAM fluorescent dyes.** Panels indicated data from 'Fenban' (FB) and 'Kouzi Yudie' (KZYD) and their F_1_ hybrids (HB): (A) locus heterozygosities in the 'Fenban' , two alleles; (B) locus heterozygosities in the 'Kouzi Yudie', two alleles; (C) locus heterozygosities in parental lines, two alleles; (D) locus heterozygosities in parental lines, three alleles; (E) locus heterozygosities in parental lines, four alleles; (F) locus homozygosity in parental lines.
